# Pumpkin Seed for Tape Worm

**Published:** 1880-02

**Authors:** H. J. Thomas

**Affiliations:** Greenwood, Wis.


### ARTICLE XII.

### PUMPKIN SEED FOR TAPE-WORM.

*Messrs. Editors:*—Seeing in my January number of your JOURNAL AND EXAMINER, an article entitled "My Struggle with a Tape-worm," I want, through your columns, to offer Dr. Pratt my sympathy and indorse his final method. I have been in the same boat, only *a little more so.* In June last, a lady called on me with a link of tænia solium, which she said her little boy (eight years old) had passed. She said he had been passing them for over a month before she had found it out.

Now what was strange to me in this case, was the fact, that links of the worm would come away while the child was at his play or at school. In fact I was at the house once, a few days after the first treatment I gave, when the boy picked a link from the back of his neck, saying "there's another of those worms."

This, it seems, was an unfrequent occurrence. The links would sometimes appear at the neck, again he would feel them on his belly and pick them off, and scarcely a night passed that he did not find joints in his drawers or stockings.

I (like Dr. Pratt) used fld. ext. male fern as largely as I dared (after fasting), and gave castor oil as is customary. No especial results. Then used capsules of oil of male fern with kamala. Again no result. Then consulted a neighboring physician, and gave oil of male fern itself. I obtained it in Chicago of a druggist, who assured me it was of prime quality. For the purgative I gave a mixture as follows:

| | | | | |
|---|---|---|---|---|
| ℞ | Ol. terebinth........................ | (gtt. xx) | 1 | |
| | Ol. tiglii.............................. | (gtt. ij) | | 10 |
| | Ol. ricini.............................. | (f℥ss) | 16 | |
| | Muc. acacia.......................... | (f℥ss) | 16 | |

Mix.

Now, I was sure of the offender. The result was half a dozen copious stools with some griping and tenesmus toward the last, but *no worm.* Not even as many links as would pass in ordinary stools. About this time the mother was advised by an "old lady" to give pumpkin seeds. She asked me about using them, but I discouraged the idea, saying in a few days I would give another preparation which I was sure would bring the worm. But in the interval they tried the pumpkin seeds, and the boy came running into my office one day, breathless and excited, saying, "Oh, doctor, we've got the worm," "Come and see it!"

I found he had passed a *complete* worm about twenty feet long. Imagine my chagrin when "Young America," in an exulting way said, "Oh, doctor, grandma's pumpkin seed beat all your medicines." I had to confess it did. But soon the boy again began to pass joints of another worm, which were broader than any passed before. I advised a repetition of the pumpkin seed, and the result was a worm about forty feet long. Since then (last September), the boy has been in the best of health. I kept him for two months on sulph. of iron (j gr.) .05 doses morning and night), and tr. colomb. and gentian co. before meals.

H. J. THOMAS, M.D.

GREENWOOD, WIS., Jan. 16, 1880.

